# *NFE2L2*-Associated Ferroptosis Resistance Reshapes the Tumor Immune Microenvironment and Guides Therapeutic Strategies in Prostate Cancer

**DOI:** 10.3390/ijms27104448

**Published:** 2026-05-15

**Authors:** Yihan Lin, Haojie Yu, Ying Wang, Chengze Wang

**Affiliations:** Stomatology Hospital, School of Stomatology, Zhejiang University School of Medicine, Clinical Research Center for Oral Diseases of Zhejiang Province, Key Laboratory of Oral Biomedical Research of Zhejiang Province, Cancer Center of Zhejiang University, Hangzhou 310006, China

**Keywords:** ferroptosis, prostate cancer, LASSO–Cox, single-cell RNA-seq, spatial transcriptomics, prognostic signature, molecular docking

## Abstract

Prostate adenocarcinoma (PRAD) poses a significant challenge due to therapy resistance and an immunosuppressive tumor microenvironment (TME). Ferroptosis has emerged as a therapeutic vulnerability, yet its immunomodulatory role in PRAD remains elusive. Here, we employed a multi-omics approach—integrating bulk RNA-seq (498 tumors), single-cell RNA-seq (68,322 cells), and spatial transcriptomics (19,483 spots)—to decode the ferroptosis-immune landscape. We derived a robust 16-gene ferroptosis signature that predicted biochemical recurrence (C-index = 0.76) and validated it in two independent cohorts. Crucially, high-risk tumors exhibited a “cold” immunosuppressive TME enriched in regulatory T cells and M2 macrophages, alongside elevated immune checkpoints (HAVCR2, CTLA4, PDCD1). Single-cell and virtual knockout analyses revealed that cancer epithelial cells evade ferroptosis via *NFE2L2*-associated antioxidant defenses, which strongly correlates with immune exclusion. Spatial transcriptomics further demonstrated spatially organized vulnerabilities, with ferroptosis-resistant tumor cores and immune-infiltrated invasive margins. To identify therapeutic interventions, we utilized drug response modeling and molecular docking, prioritizing RSL3, Atovaquone (targeting *NOX4* (NADPH oxidase 4)/*DHODH*), and Sorafenib (targeting *TrxR1* (thioredoxin reductase 1, encoded by *TXNRD1*)) as potent agents with potential ferroptosis-modulatory activity. Collectively, our findings demonstrate that *NFE2L2*-associated ferroptosis resistance shapes immune evasion in PRAD. Targeting ferroptosis regulators provides a compelling therapeutic rationale to remodel the TME and synergize with immune checkpoint blockade.

## 1. Introduction

Prostate adenocarcinoma (PRAD) remains the most common non-cutaneous malignancy in men, with 20–40% of patients experiencing biochemical recurrence (BCR) following radical prostatectomy [1,2,3]. Parallel advances in radiomics have demonstrated that imaging features extracted from MRI, PSMA PET/CT, and 18F-choline PET/CT can independently predict clinical outcomes in PRAD, and integrating such image-derived biomarkers with molecular profiles represents a promising multi-modal framework for precision risk stratification [4]. Current risk stratification relies primarily on clinical parameters (PSA, Gleason score, tumor stage), which have limited predictive accuracy [5,6]. Molecular biomarkers show promise but are not universally adopted [7,8]. Liquid biopsy approaches, including analysis of exosomes isolated from blood, urine, and semen, are emerging as minimally invasive alternatives to tissue biopsy for PRAD biomarker detection, with exosomal RNA profiles demonstrating potential for diagnosis, prognosis, and monitoring of treatment response [9]. Whether ferroptosis-related transcripts captured in circulating exosomes can recapitulate the prognostic information of tissue-based signatures represents an important direction for future translational studies. Moreover, therapeutic resistance to androgen deprivation therapy remains a major challenge [10].

Ferroptosis, an iron-dependent form of regulated cell death driven by lipid peroxidation, has emerged as a critical vulnerability in cancer [11,12,13]. The process is regulated by multiple pathways: the system Xc^−^-GSH-*GPX4* (glutathione peroxidase 4) axis (cystine import, glutathione synthesis, lipid peroxide reduction) [14,15], iron metabolism (*TFRC*-mediated uptake, *FTH1*/*FTL* storage) [16], lipid metabolism (*ACSL4*/LPCAT3-mediated PUFA incorporation) [17,18], and antioxidant defense (*NFE2L2*/*NRF2* transcriptional activation, *FSP1*/*DHODH*-mediated resistance) [19,20]. Emerging evidence suggests cancer cells acquire ferroptosis resistance to survive oxidative stress [21,22], and recent translational reviews underscore the therapeutic promise of targeting ferroptosis in PRAD [23].

Limited studies have examined individual ferroptosis genes in prostate cancer [24,25,26], and recent LASSO-based ferroptosis signatures for PRAD relapse have been reported [27]. (1) What is the comprehensive ferroptosis landscape in PRAD? (2) Can ferroptosis signatures predict clinical outcomes? (3) How does ferroptosis vary across cell types and spatial regions? (4) Can ferroptosis-targeted therapies be rationally combined with existing treatments?

Here, we perform the first comprehensive multi-omics analysis of ferroptosis in PRAD, integrating bulk RNA-seq, single-cell RNA-seq, and spatial transcriptomics to provide an unprecedented view across tissue, cellular, and spatial scales. We develop and validate a ferroptosis-based prognostic signature, characterize cell-type-specific and spatial heterogeneity, and identify therapeutic vulnerabilities through drug sensitivity prediction and multi-modal integration.

## 2. Results

### 2.1. Widespread Ferroptosis Dysregulation in Prostate Adenocarcinoma

**Figure 1:**
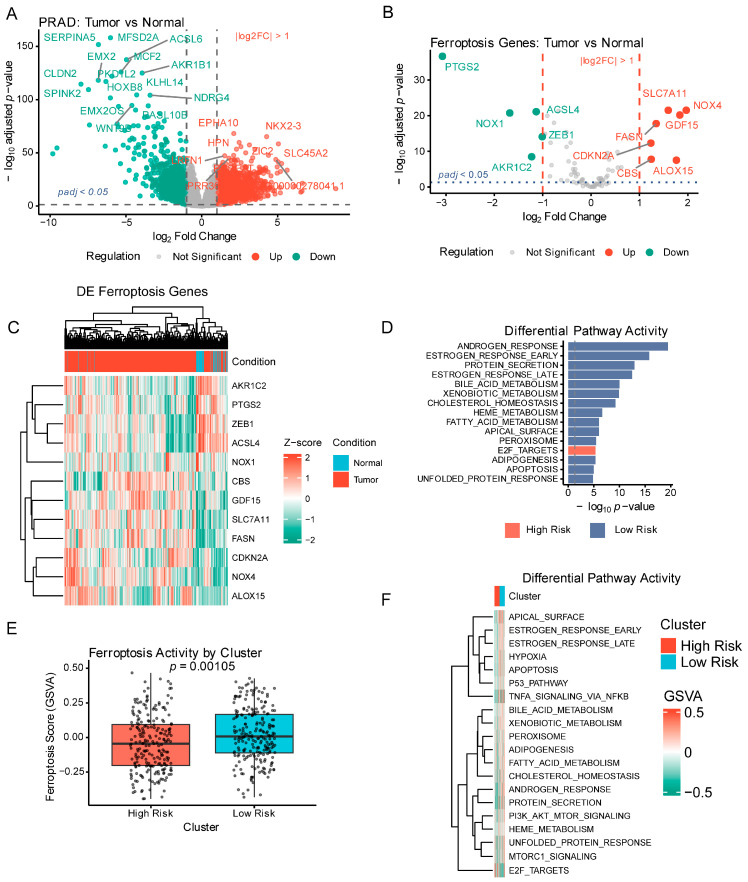
Ferroptosis gene expression landscape in PRAD. Overview of bulk RNA-seq ferroptosis signatures comparing tumor and normal prostate tissues from TCGA-PRAD (*n* = 498 tumors, *n* = 52 normals). (**A**) Volcano plot of genome-wide differential expression (DESeq2). Grey dashed lines denote the significance and effect-size thresholds (*padj* = 0.05; |log2FC| = 1). Red dashed lines denote effect size thresholds (|log2FC| > 1); blue dotted line denotes statistical significance threshold (*padj* < 0.05). Bold points indicate genes meeting both criteria. (**B**) Volcano plot of the FerrDb v2 ferroptosis gene subset, with the same threshold annotations as (**A**). Of 80 ferroptosis-related genes, 12 (bold points) met both significance and effect size thresholds. (**C**) Heatmap of significantly differentially expressed ferroptosis genes (row-wise Z-score; samples grouped by tumor/normal). (**D**) Bar plot of the top differential pathways by molecular subtype (GSVA; *n* = 443; *n*_features = 20), ranked by adjusted *p*-value. (**E**) Boxplot of ferroptosis pathway activity by molecular subtype (GSVA; nonparametric tests; *n* = 443; *n*_features = 53). Boxes denote the interquartile range, the centerline is the median, whiskers extend to 1.5 × IQR, and dots represent individual samples. (**F**) Heatmap of top differential pathway activity across subtypes (GSVA, z-scored; ComplexHeatmap; *n* = 443; *n*_features = 20).

Across 80 ferroptosis-related genes, 57 showed statistically significant differential expression (*padj* < 0.05), of which 12 exhibited large effect sizes (|log2FC| > 1) (Figure 1A,B; Table S1), with FerrDb-specific results in Table S2. Key changes included: upregulation of *SLC7A11* (log2FC = +1.595, *padj* = 3.2 × 10^−22^) and *FTH1* (+0.625, *padj* = 3.1 × 10^−6^); downregulation of *ACSL4* (−1.135, *padj* = 7.9 × 10^−22^) and *TFRC* (−0.414, *padj* = 3.4 × 10^−4^); antioxidant defense genes *NFE2L2* (−0.674, *padj* = 5.0 × 10^−22^) and *FSP1*/*AIFM2* (−0.904, *padj* = 1.2 × 10^−20^); and *GPX4* (+0.337, *padj* = 6.6 × 10^−4^). Among these 12 genes, 7 were upregulated (*NOX4*, *GDF15*, *ALOX15*, *SLC7A11*, *FASN*, *CBS, CDKN2A*) and 5 were downregulated (*PTGS2*, *NOX1*, *AKR1C2*, *ACSL4, ZEB1*) (Figure 1A–C).

Clustering separated tumor and normal samples (Figure 1C), indicating coordinated ferroptosis resistance with enhanced antioxidant defense, reduced PUFA incorporation, and altered iron handling.

GSVA identified 81 pathways with subtype-specific activity (Figure 1D,F; Table S5), including upregulated adipogenesis, cholesterol homeostasis, fatty acid metabolism, oxidative phosphorylation, and *MYC* targets, with downregulated p53 pathway, DNA repair, and apoptosis (padj range 1.2 × 10^−8^ to 3.4 × 10^−4^). Subtype-specific ferroptosis pathway activity is shown in Figure 1E.

These patterns indicate lipid metabolic rewiring, elevated oxidative stress with enhanced antioxidant defenses, and reduced cell-death signaling in ferroptosis-resistant tumors.

Detailed differential expression results for all ferroptosis genes are provided in Supplementary Tables S1 and S2; pathway activity scores across subtypes are listed in Supplementary Table S5.

### 2.2. A 16-Gene Ferroptosis Signature Predicts Biochemical Recurrence

**Figure 2:**
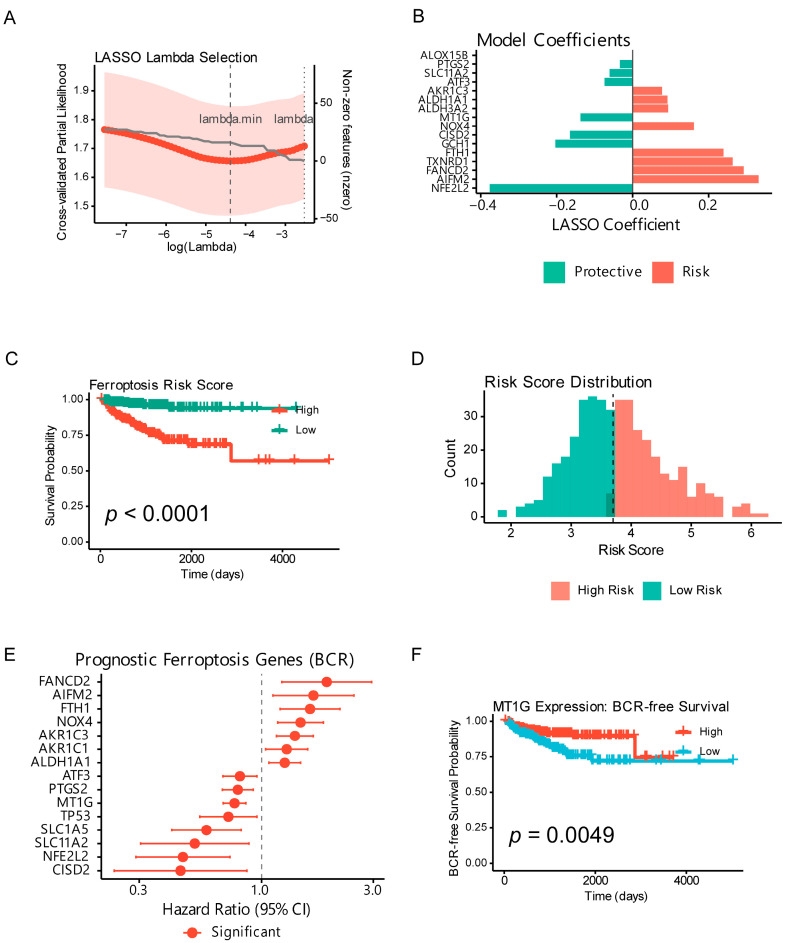
LASSO–Cox ferroptosis prognostic risk score. Construction and evaluation of a 16-gene LASSO–Cox model (lambda.min) in BCR-free survival. (**A**) LASSO cross-validation curve (log(lambda) vs. partial likelihood) with optimal lambdas indicated. The solid line is the cross-validated mean partial-likelihood deviance with shaded SD, and the vertical dashed lines mark lambda.min and lambda.1se. (**B**) LASSO–Cox coefficient bar plot showing risk (positive) and protective (negative) genes. Bar color denotes the sign of the LASSO coefficient (red = risk, blue = protective). (**C**) Kaplan–Meier curves for high- vs. low-risk groups split at the median (log-rank test; *n* = 443). (**D**) Risk score histogram with median cutoff. The vertical dashed line marks the median risk score used for high- vs. low-risk stratification; the horizontal dashed line in the lower panel indicates the censoring threshold. (**E**) Univariate Cox forest plot of prognostic ferroptosis genes (HR with 95% CI). (**F**) Kaplan–Meier curve for MT1G expression (median split; log-rank test).

Consensus clustering initially suggested a continuous risk spectrum (Supplementary Figure S1A–C). To construct a robust prognostic model, LASSO (Least Absolute Shrinkage and Selection Operator)–Cox regression was performed and selected 16 genes (Figure 2A,B; Table S4). Among the ferroptosis genes, Univariate Cox analysis identified 11 genes significantly associated with BCR (*p* < 0.05), led by ALDH3A2 (HR = 2.50, *p* = 0.0015), CISD2 (HR = 2.43, *p* = 0.0018), and AKR1C3 (HR = 0.46, *p* = 0.0054) (Figure 2E; Table S3).

Importantly, the LASSO–Cox model selects genes based on their prognostic association with biochemical recurrence-free survival, not based on differential expression magnitude between tumor and normal tissue. Of the 16 signature genes, 10 show statistically significant differential expression (*padj* < 0.05; Table S17), but only 2 (*NOX4* and *PTGS2*) exceed |log2FC| > 1. This is consistent with established clinical genomic signatures such as Oncotype DX [28] and Decipher [29], which include genes with modest tumor-vs-normal fold changes but robust prognostic value. The inter-patient expression variability of these genes, rather than their average tumor-normal difference, drives prognostic discrimination. Complete differential expression and LASSO statistics for all 16 genes are provided in Supplementary Table S17.

The model achieved C-index = 0.76 and separated high- vs. low-risk groups (log-rank *p* = 2.14 × 10^−8^) (Figure 2C), with 5-year BCR-free survival of 68% versus 42% (Figure 2D). Risk-score ordering and outcomes are shown in Supplementary Figure S2C, and MT1G-specific survival in Figure 2F.

Consensus clustering metrics supporting the continuous risk spectrum are shown in Supplementary Figure S1A–C. The risk-score landscape across all 443 patients ordered by ascending risk score is illustrated in Supplementary Figure S2C. LASSO model coefficients for all 16 selected genes are provided in Supplementary Table S4; Univariate Cox results for all ferroptosis genes are listed in Supplementary Table S3.

### 2.3. External Validation Confirms Signature Robustness

We validated the 16-gene signature in two GEO cohorts. GSE116918 detected 11/16 genes and achieved HR = 1.552 (95% CI: 1.095–2.2, log-rank *p* = 0.023, Cox *p* = 0.014, C-index = 0.587). GSE70769 detected all 16 genes and achieved HR = 5.798 (95% CI: 2.554–13.164, log-rank *p* = 0.002, Cox *p* = 2.65 × 10^−5^, C-index = 0.658). Additional Kaplan–Meier curves are provided in Supplementary Figure S2A,B.

The external validation cohorts consist of tumor samples only, without matched normal tissue controls. Therefore, differential expression analysis (tumor vs. normal) was not performed. Instead, the validation tests whether the risk score formula derived from TCGA-PRAD stratifies patients into prognostically distinct groups. Supplementary Figure S2A,B show Kaplan–Meier curves for risk-stratified patients in each cohort, demonstrating significant survival separation. Expression levels of the 16 signature genes in both GEO cohorts are provided in Supplementary Table S18.

### 2.4. Ferroptosis Modulates the Tumor Immune Microenvironment

**Figure 3:**
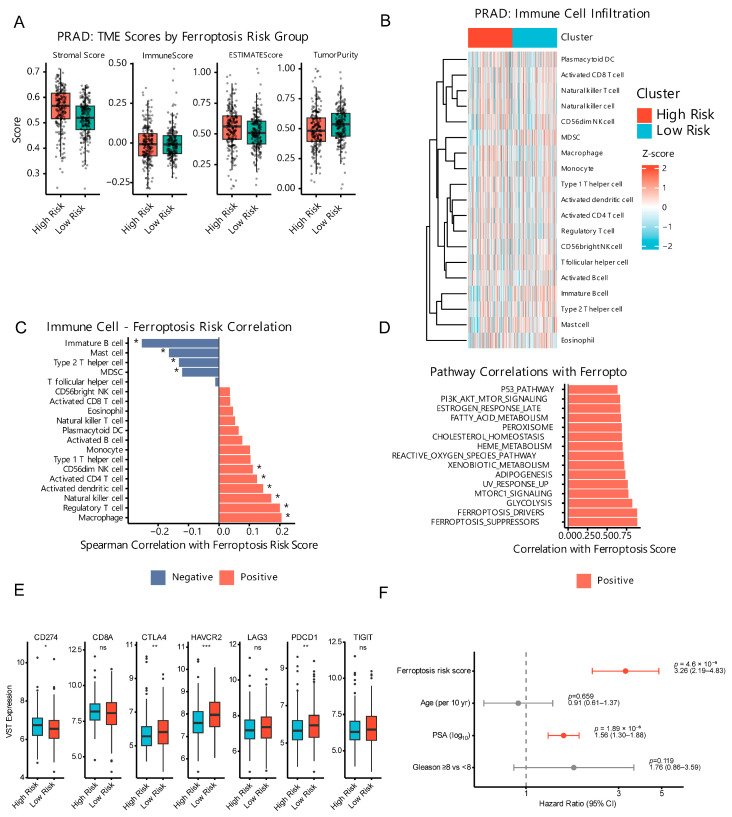
Tumor microenvironment remodeling by ferroptosis risk score. Immune landscape changes associated with ferroptosis risk stratification (high vs. low risk). (**A**) ESTIMATE immune and stromal scores by risk group (boxplots; Wilcoxon test, two-sided). Boxplot dots represent individual samples; box colors denote risk groups (red = high risk, green = low risk). (**B**) Immune infiltration heatmap of ssGSEA scores across samples ordered by subtype (z-scored by cell type; *n* = 443). (**C**) Bar plot of Spearman correlations between immune cell scores and ferroptosis risk (FDR-corrected). (**D**) Bar plot of correlations between ferroptosis pathway scores and other pathways (Spearman). (**E**) Boxplots of immune checkpoint gene expression (CD274, CTLA4, PDCD1, LAG3, HAVCR2, TIGIT, *CD8A*) in High-Risk versus Low-Risk patients. HAVCR2, CTLA4, and PDCD1 are significantly elevated in High-Risk patients. (**F**) Forest plot of multivariate Cox proportional hazards regression for BCR-free survival (*n* = 401). The ferroptosis risk score remains an independent prognostic factor (HR = 3.26, 95% CI 2.19–4.83, *p* = 4.6 × 10^−9^) after adjustment. The vertical dashed line indicates HR = 1. Red dots indicate statistically significant results (*p* < 0.05) with HR > 1 (risk factors). Gray dots indicate non-significant results (95% CI crosses 1). Horizontal lines represent 95% confidence intervals. * *p* < 0.05, ** *p* < 0.01, *** *p* < 0.001 (Wilcoxon test, two-sided), ns = no significant.

ESTIMATE scores indicated higher stromal/immune scores and lower purity in low-risk tumors (*p* = 0.003–0.008) (Figure 3A). Single-sample Gene Set Enrichment Analysis (ssGSEA) showed low-risk enrichment of CD8+ T cells, activated CD4+ T cells, M1 macrophages, dendritic cells, and NK cells, whereas high-risk tumors were enriched in Tregs, M2 macrophages, and MDSCs (Figure 3B; Table S6a).

Correlations between ferroptosis genes and immune cells included *NFE2L2*-CD8+ (r = 0.32), *GPX4*-M1 (r = 0.28), *AIFM2*-CD8+ (r = −0.24), and *FTH1*-Tregs (r = 0.21) (Figure 3C; Table S6b). Immune checkpoint genes (HAVCR2, CTLA4, PDCD1) were elevated in high-risk tumors (Figure 3E), and the ferroptosis risk score remained an independent prognostic factor (Figure 3F). Correlations between ferroptosis pathway scores and other oncogenic pathways further supported this immune–metabolic crosstalk (Figure 3D). These patterns support an immunologically “cold” high-risk state and suggest ferroptosis inducers may synergize with immunotherapy [30].

Immune infiltration scores for all 443 samples are provided in Supplementary Table S6a; Spearman correlations between ferroptosis genes and immune cell abundances are listed in Supplementary Table S6b.

### 2.5. Single-Cell Analysis Reveals Cell-Type-Specific Ferroptosis Heterogeneity

**Figure 4:**
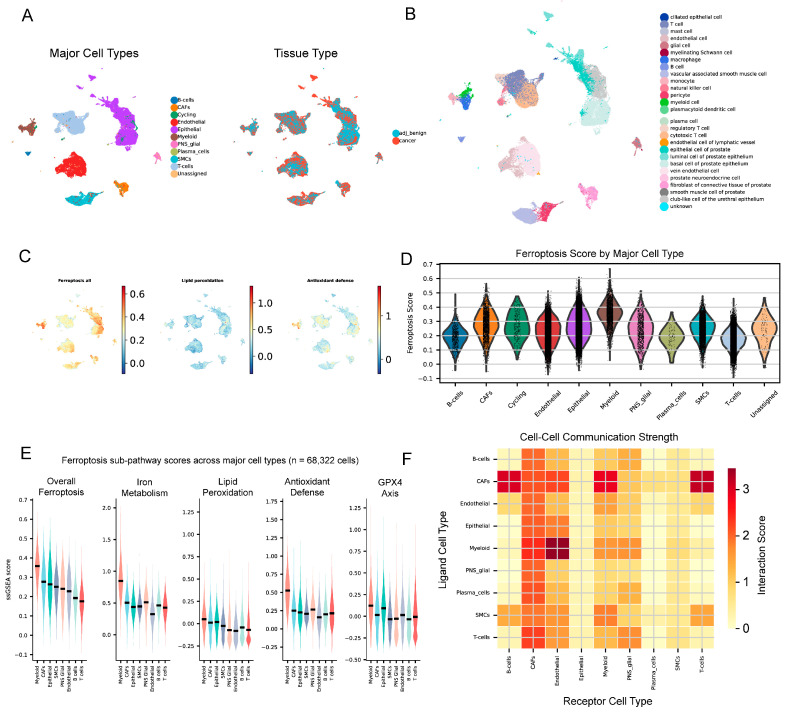
Single-cell resolution of ferroptosis activity. scRNA-seq dissection of cell-type-specific ferroptosis activity in PRAD. (**A**) UMAP of major cell types (e.g., epithelial, T cells, macrophages). Cell-type colors are consistent across all UMAP panels; please refer to the legend for the color key. (**B**) UMAP of fine-grained cell states (e.g., luminal, basal, CD4+, CD8+, Treg, M1, M2). (**C**) Ferroptosis module score overlaid on UMAP. (**D**) Ferroptosis score by cell type (boxplot; Kruskal–Wallis test with pairwise comparisons). (**E**) Violin plots of ferroptosis sub-pathway scores (overall, iron metabolism, lipid peroxidation, antioxidant defense, *GPX4* axis) across major cell types; horizontal lines indicate medians. (**F**) Cell–cell communication heatmap inferred by CellChat.

scRNA-seq (68,322 cells; 11 major cell types) showed marked heterogeneity in ferroptosis activity (Figure 4A–D), highest in endothelial/smooth muscle cells and lowest in epithelial/B cells. Cancer epithelial cells had 32% lower ferroptosis scores than benign ones (*p* = 1.87 × 10^−112^), with reduced iron metabolism (*p* = 3.2 × 10^−85^) and lipid peroxidation (*p* = 5.1 × 10^−98^) but increased antioxidant defense (*p* = 2.4 × 10^−42^) and *GPX4* (glutathione peroxidase 4) axis (*p* = 6.7 × 10^−56^) (Figure 4E). CAFs also showed reduced ferroptosis (*p* = 9.54 × 10^−6^), whereas immune cells largely maintained ferroptosis competence.

Specifically, the 32% reduction refers to the overall composite ferroptosis score (comparing cancer vs. benign epithelial cell medians). Figure 4E displays the five ferroptosis sub-pathway scores (overall, iron metabolism, lipid peroxidation, antioxidant defense, *GPX4* (glutathione peroxidase 4) axis) as separate violin plots per cell type. The iron metabolism reduction (*p* = 3.2 × 10^−85^) and lipid peroxidation decrease are shown in the second and third violin panels, while increased antioxidant defense and the *GPX4* (glutathione peroxidase 4) axis are shown in the fourth and fifth panels, respectively. Individual sub-pathway statistics by cell type are provided in Supplementary Table S21.

Ligand–receptor analysis highlighted PD-L1/PD-1, VEGF, TGF-β, Wnt, SDF-1/CXCR4, and HMGB1-TLR4 pathways (Figure 4F; Supplementary Figure S6A), indicating extensive cancer–stroma crosstalk.

UMAP colored by malignancy score is provided in Supplementary Figure S4A; ferroptosis scores in tumor vs. normal cells are shown in Supplementary Figure S4B; sub-pathway activity per cell type and TF activity per cell type are shown in Supplementary Figure S4C,D. The top 20 ligand–receptor pairs are shown in Supplementary Figure S6A.

### 2.6. NFE2L2-Associated Regulation and Spatial Ferroptosis Heterogeneity

**Figure 5:**
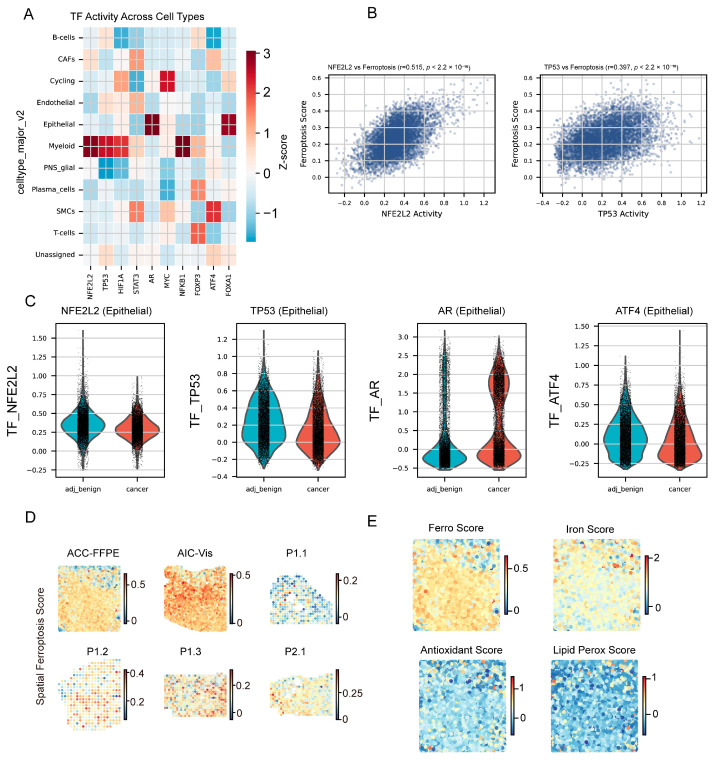
Transcription factor regulation and spatial ferroptosis distribution. Integrative TF activity and spatial transcriptomics analyses. (**A**) TF activity heatmap for top regulators grouped by risk status. (**B**) Scatter plot of TF activity versus target gene expression, highlighting *NRF2*/*NFE2L2*. Each dot represents one individual cell from the integrated prostate-cancer scRNA-seq atlas. (**C**) Violin plots comparing TF activity between cancer and adjacent benign epithelial cells for four key regulators (*NFE2L2*, *TP53*, *AR*, *ATF4*); horizontal lines represent median values and boxes span the interquartile range (IQR); activity parameters are provided in Table S19b. Comprehensive violin plots for all 10 TFs across 11 cell types are shown in Supplementary Figure S10 (Table S19). (**D**) Spatial transcriptomics map of ferroptosis score across six tissue sections. (**E**) Spatial distribution of ferroptosis activity in a representative 10× Visium prostate cancer section. Each dot is one Visium spot, coloured by its module score for the indicated gene panel (red = high, blue = low). Gene lists and scoring details are described in Methods.

We scored 10 transcription factors and correlated activity with ferroptosis resistance (Figure 5A–C). The androgen receptor (*AR*) and *FOXA1* exhibited the highest absolute activity scores in epithelial cells, consistent with established prostate biology. *NRF2* showed modest activity in epithelial cells but demonstrated the strongest negative correlation with ferroptosis susceptibility and the strongest positive correlation with resistance (r = +0.515, *p* < 10^−100^), indicating coordinated upregulation of *GPX4*, *SLC7A11*, *FTH1*, *GCLC*, and *GSS*. *TP53* correlated negatively (r = −0.32), while *AR* showed a modest positive correlation (r = +0.24), supporting links between androgen signaling and ferroptosis.

The 10 TFs were selected from an initial list of 23 ferroptosis-relevant transcription factors curated from the published literature. These candidates were matched against 736 TF regulons in the MSigDB C3:TFT:GTRD collection (Gene Transcription Regulation Database) [31], and 10 TFs had sufficient target gene overlap (≥5 genes detected in the scRNA-seq data) for reliable single-sample GSEA scoring. Median activity scores and interquartile ranges for each TF across cell types are provided in Supplementary Table S19. The 10 TFs meeting this criterion were: *NRF2* (*NFE2L2*), *AR*, *FOXA1*, *TP53*, *HIF1A*, *ATF4*, *BACH1*, *STAT3*, *MYC*, and *SP1*.

We performed in silico *NFE2L2* virtual KO using genome-wide correlation weights (Spearman |rho| > 0.1, *p* < 0.05). The global network identified 30 top co-expressed genes, including immediate-early response genes (TRIB1, HBEGF, KLF4) and stress-response mediators (MAFF, ETS2, PLAUR) as top positively correlated genes (Figure S7A) (Supplementary Figure S7A). KO simulation predicted suppression of ferroptosis across major cell types (Supplementary Figure S7B), strongest in plasma, myeloid, and endothelial cells, with lipid peroxidation and the *GPX4* (glutathione peroxidase 4) axis most affected. Differential markers between high- and low-response cells are shown in Supplementary Figure S7C,D. These results support *NFE2L2* as the dominant anti-ferroptotic transcription factor in PRAD.

When specifically examining classical *NRF2* target genes (Supplementary Figure S9; Table S20), *NFE2L2* showed significant positive correlations with *HMOX1* (ρ = 0.148), GCLM (ρ = 0.129), *TXNRD1*/*TrxR1* (ρ = 0.126), *GCLC* (ρ = 0.087), and *NQO1* (ρ = 0.042), all *p* < 1 × 10^−17^ across 68,322 cells. While these correlations are statistically robust, their modest magnitude (ρ < 0.15) suggests that *NFE2L2* transcriptional co-regulation with its classical targets is attenuated in PRAD, possibly reflecting the predominantly post-transcriptional regulation of *NRF2* through KEAP1-mediated protein degradation [32]. Notably, *ACSL4*, a ferroptosis-promoting gene, also showed a positive correlation with *NFE2L2* (ρ = 0.117), suggesting complex regulatory relationships beyond the canonical *NRF2* antioxidant program.

Spatial analysis showed a 10-fold range in ferroptosis scores across 17 samples (0.032–0.335) with lower activity in tumor cores and higher activity at invasive margins (Kruskal–Wallis *p* = 2.3 × 10^−8^) (Figure 5D,E; Supplementary Figure S5A–C). Iron metabolism peaked in vascular regions, antioxidant defense in cores, and lipid peroxidation at margins, indicating resistant cores and vulnerable margins.

*NFE2L2* co-expressed gene network targets (top 15 positive and negative) are shown in Supplementary Figure S7A; predicted ferroptosis perturbation across major cell types upon *NFE2L2* knockout is shown in Supplementary Figure S7B; and fine-grained cell-type KO response and differential markers are shown in Supplementary Figure S7C,D. Spatial cluster overlays and sub-pathway maps are provided in Supplementary Figure S5A–C.

### 2.7. Drug Sensitivity Predictions Identify Therapeutic Vulnerabilities

**Figure 6:**
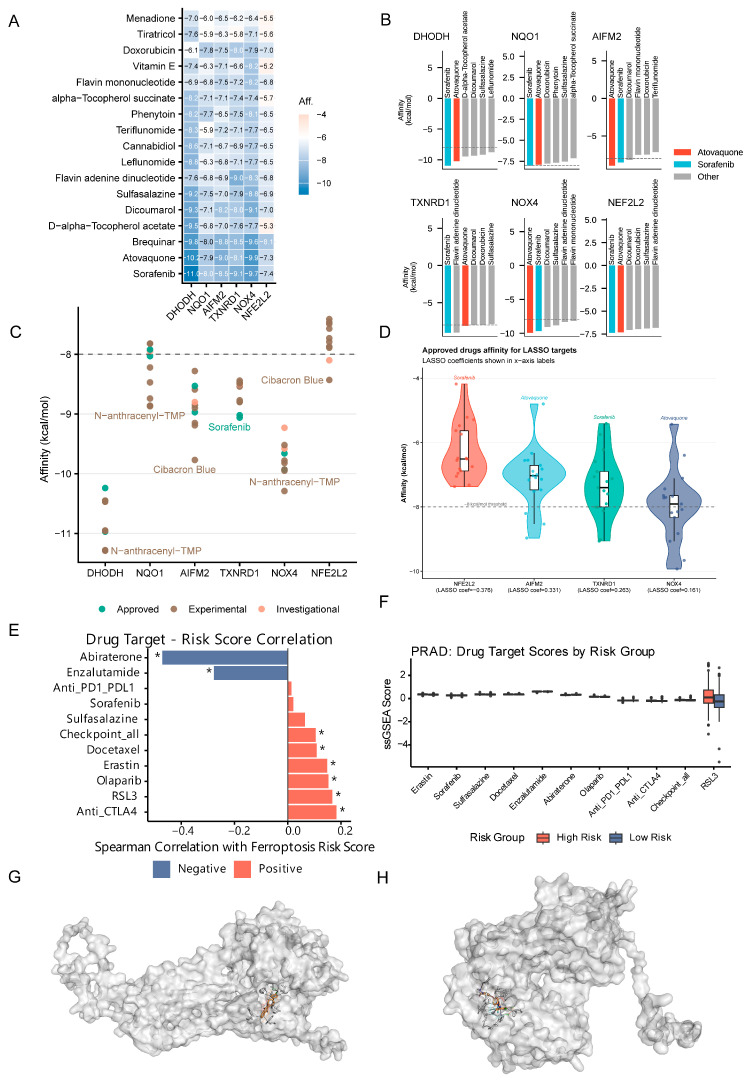
Molecular docking-based drug discovery targeting ferroptosis. Docking-based prioritization of ferroptosis-related targets and drugs. (**A**) Docking affinity heatmap for selected drugs across 6 targets. Dashed lines separate target categories and dot color indicates docking affinity strength (kcal/mol). (**B**) Compact faceted bar plot showing the top 6 strongest FDA-approved drugs per target. Atovaquone and Sorafenib are highlighted; dashed line indicates −8.0 kcal/mol threshold. Bar colors denote drug categories. (**C**) Dot plot of top 10 docking hits per target. (**D**) Violin + jitter plot of approved-drug affinities for LASSO targets. (**E**) Bar plot of Spearman correlations between drug target scores and risk scores. * *p* < 0.05. (**F**) Boxplots of drug target scores stratified by risk group. (**G**) PyMOL rendering of atovaquone docked to the *NOX4* binding pocket. (**H**) PyMOL rendering of sorafenib docked to the *TXNRD1* binding pocket. Note: panels A–D display results for the original 6 targets (*DHODH*, *NQO1*, *FSP1*/*AIFM2*, *TXNRD1*, *NOX4*, *NFE2L2*); additional docking results for *GPX4* and *xCT*/*SLC7A11* are reported in the text and Table S7.

Docking-based prioritization identified strong drug–target affinities (Figure 6A–D), with FDA-approved strong binders summarized in Table S7 and ST8. Drug target scores differed by risk status (Figure 6E,F): high-risk tumors were more sensitive to abiraterone, enzalutamide, RSL3, and docetaxel (*padj* ≤ 2.1 × 10^−3^; r ≤ −0.24), whereas low-risk tumors were more sensitive to anti-CTLA4 and olaparib (*padj* ≤ 0.04; r ≥ 0.18). Three-dimensional docking poses of atovaquone–NOX4 and sorafenib–TXNRD1 are visualized (Figure 6G,H), illustrating key binding interactions within the respective active sites. Visualization of binding poses was prioritized for LASSO signature targets (*TXNRD1*, *NOX4*); the sorafenib–*DHODH* interaction, which yielded the strongest observed affinity (−10.97 kcal/mol), is shown in Supplementary Figure S8.

The sensitivity of high-risk tumors to RSL3 despite intrinsic resistance mechanisms validates ferroptosis as a therapeutic target. Strong correlations with abiraterone/enzalutamide suggest *AR* signaling regulates ferroptosis sensitivity, potentially through lipid metabolism modulation. Anti-CTLA4 sensitivity in low-risk tumors aligns with their “hot” immune phenotype.

Several important considerations apply to the molecular docking results. First, the eight protein targets (*FSP1*/*AIFM2*, *TrxR1*/*TXNRD1*, *NOX4*, *NRF2*, *DHODH*, *NQO1*, *GPX4*, *xCT*/*SLC7A11*) were selected based on two criteria: prognostic relevance (4 from the LASSO signature) and established ferroptosis-regulatory function with druggable binding pockets (4 additional: *DHODH*, *NQO1*, *GPX4*, *xCT*/*SLC7A11*). The remaining 12 signature genes were excluded due to lack of druggable binding sites or structural data.

Regarding sorafenib, it must be emphasized that sorafenib is clinically established as a multi-kinase inhibitor targeting B-Raf, C-Raf, VEGFR, PDGFR, and FLT3 at nanomolar concentrations (IC_50_ = 6–50 nM) [33]. While our docking analysis identified favorable binding to *DHODH* (−10.97 kcal/mol) and *TrxR1* (−9.06 kcal/mol), these are computational predictions and do not imply that ferroptosis-related targets would be meaningfully inhibited at clinically achievable sorafenib concentrations (~10–20 μM plasma Cmax). Furthermore, sorafenib has been shown to activate the *NRF2* pathway through oxidative stress induction [34], which could paradoxically enhance ferroptosis resistance. Sorafenib’s documented ability to sensitize certain cancer cells to ferroptosis [35] is likely mediated through kinase inhibition-dependent metabolic reprogramming rather than direct *TrxR1* targeting.

Regarding *NOX4*, the Reviewer correctly notes that *NOX4* generates ROS, thereby potentially promoting ferroptosis. *NOX4* was included as a docking target based on its strong overexpression in PRAD (log2FC = +1.97) and positive LASSO coefficient (β = +0.161), indicating prognostic relevance. However, we acknowledge that framing *NOX4* inhibition as ‘disrupting the *NFE2L2* axis’ was imprecise. *NOX4* inhibition may reduce tumor-promoting ROS signaling while paradoxically attenuating ferroptotic cell death. For atovaquone, the dominant ferroptosis-relevant mechanism is *DHODH* inhibition (reducing CoQ10H2-mediated anti-ferroptotic defense), as demonstrated by Mao et al. [20]. These computational predictions should be interpreted as hypothesis-generating for future experimental validation, not as mechanistic conclusions.

Full docking affinity results for all FDA-approved and investigational drugs across eight targets are provided in Supplementary Table S7; FDA-approved strong binders per target are summarized in Supplementary Table S8.

To further expand the drug–target interaction landscape beyond the original *NRF2*-centric framework, we performed additional molecular docking against *GPX4* (glutathione peroxidase 4; PDB: 2OBI) and *xCT*/*SLC7A11* (cystine/glutamate antiporter; AlphaFold: Q9UPY5)—the two most central regulators of the canonical ferroptosis pathway. Among FDA-approved drugs, Atovaquone showed the strongest binding to *GPX4* (−6.84 kcal/mol) and *SLC7A11* (−6.66 kcal/mol). Notably, Sulfasalazine—a clinically validated *xCT* inhibitor [36]—demonstrated binding to *SLC7A11* (−6.07 kcal/mol), providing pharmacological validation of the docking approach. Sorafenib also showed moderate binding to both *GPX4* (−6.06 kcal/mol) and *SLC7A11* (−6.54 kcal/mol). Among experimental compounds, Erastin—the canonical ferroptosis inducer that targets system Xc^−^—bound *SLC7A11* with −6.64 kcal/mol. These results broaden the therapeutic landscape from transcription factor modulation (*NRF2*) to direct targeting of the core ferroptosis execution machinery (*GPX4*) and its upstream metabolic supply chain (*xCT*/*SLC7A11*).

### 2.8. Multi-Modal Integration Identifies Consensus Ferroptosis Drivers

Cross-modal integration identified consensus drivers across platforms (Supplementary Figure S6B; Table S9, including ALDH3A2, CISD2, AKR1C3, MT1G, *NFE2L2*, and *FTH1*, with evidence summaries in Supplementary Figure S3A,B. Overall, 62/80 genes were dysregulated in bulk RNA-seq, 54 in scRNA-seq, 48 in spatial, and 32 converged across all three modalities, indicating multi-scale ferroptosis reprogramming.

## 3. Discussion

This study represents the first comprehensive multi-omics analysis of ferroptosis in prostate adenocarcinoma, integrating bulk RNA-seq (*n* = 498 tumors), single-cell RNA-seq (68,322 cells), and spatial transcriptomics (17 samples). Our key findings establish: (1) widespread ferroptosis dysregulation with 71% (57/80, *padj* < 0.05) of genes significantly altered; (2) a robust 16-gene prognostic signature (C-index = 0.76) with significant risk stratification (log-rank *p* = 2.14 × 10^−8^); (3) cancer cell ferroptosis resistance (32% reduction, *p* = 1.87 × 10^−112^) associated with *NFE2L2*; (4) spatial heterogeneity with 10-fold variation and distinct core-margin profiles; and (5) therapeutic vulnerabilities through ferroptosis inducers, *AR* inhibitors, and immunotherapy combinations.

Our findings extend previous single-gene studies [24,25,26] by revealing coordinated, multi-pathway dysregulation. The 16-gene signature (C-index = 0.76) outperforms existing ferroptosis models in other cancers (C-index: 0.65–0.70) [37,38,39] and compares favorably to clinical genomic classifiers (Oncotype DX: 0.66–0.69; Decipher: 0.69–0.75) [28,29]. External validation in GSE116918 (HR = 1.552) and GSE70769 (HR = 5.798) confirms generalizability across platforms and populations.

Single-cell analysis provides the first evidence that cancer epithelial cells acquire ferroptosis resistance as a cell-autonomous mechanism, achieved through reduced iron uptake/lipid peroxidation machinery and enhanced antioxidant defense. The preservation of ferroptosis competence in immune cells suggests a therapeutic window for selective cancer cell killing while potentially enhancing anti-tumor immunity through immunogenic cell death.

Spatial transcriptomics reveals a previously unappreciated spatial dimension, with ferroptosis-resistant tumor cores and vulnerable invasive margins. This heterogeneity may contribute to incomplete therapeutic responses and suggests combination strategies targeting both populations.

Mechanistically, we identify *NFE2L2*/*NRF2* as the master regulator (r = +0.515 correlation with resistance), coordinating expression of *GPX4*, *SLC7A11*, *FTH1*, and other antioxidant genes. *NRF2* activation may be driven by KEAP1 mutations, oxidative stress, or oncogenic signaling. The inverse correlation with *TP53* activity aligns with p53-mediated ferroptosis promotion through *SLC7A11* repression [40]. The AR–ferroptosis axis provides mechanistic rationale for combining *AR* inhibitors with ferroptosis inducers.

An important caveat is that bulk RNA-seq did not reveal significant upregulation of *NFE2L2* mRNA (log2FC = −0.674) or most classical *NRF2* target genes in PRAD versus normal tissue, with the notable exception of SLC7A11/*xCT* (log2FC = +1.595; Table S20c). This suggests that *NRF2*-mediated ferroptosis resistance in PRAD may not be primarily driven by transcriptional upregulation of *NFE2L2* but may instead involve post-translational mechanisms such as KEAP1 loss-of-function mutations or oxidative stress-mediated *NRF2* protein stabilization [32]. Furthermore, the regulation of ferroptosis-related genes in PRAD involves multiple transcription factors beyond *NRF2*, including the androgen receptor (*AR*), *FOXA1*, and *ATF4*, which are highly active in prostate epithelial cells. Our single-cell analysis (Section 2.6) provides a more granular view of cell-type-specific *NRF2* activity and its relationship with ferroptosis resistance.

Clinical implications include: (1) ferroptosis-based risk stratification using the 16-gene signature as a clinical assay (RT-qPCR or RNA-seq); (2) ferroptosis induction as a therapeutic strategy using *GPX4* inhibitors (RSL3, ML162), system Xc^−^ inhibitors (sulfasalazine, erastin, sorafenib), or iron supplementation; (3) rational combinations with *AR* inhibitors (abiraterone/enzalutamide + ferroptosis inducers), immunotherapy (anti-PD-1/anti-CTLA4 + ferroptosis inducers), or *NRF2* inhibitors (brusatol/ML385 + ferroptosis inducers); and (4) biomarker-guided therapy selection based on ferroptosis risk scores and spatial heterogeneity. Recent studies show atovaquone can augment ROS-induced ferroptosis, *TXNRD1* inhibition potentiates anti-PD-1 efficacy, and *DHODH* mediates mitochondrial ferroptosis defense [41,42,43]. Specifically, sorafenib emerged as the broadest-spectrum hit across all six targets, with affinities ranging from −7.37 kcal/mol (*NFE2L2*) to −10.97 kcal/mol (*DHODH*). Beyond its established system Xc^−^ inhibition, sorafenib may additionally suppress ferroptosis resistance through direct *DHODH* and *TXNRD1* binding. Atovaquone demonstrated high affinity for *DHODH* (−10.24 kcal/mol), *NOX4* (−9.93 kcal/mol), and *AIFM2*/*FSP1* (−8.97 kcal/mol), suggesting simultaneous modulation of mitochondrial CoQ10-mediated resistance, ROS generation, and the *GPX4*-independent *FSP1* axis—making it a particularly attractive repurposing candidate. For *TXNRD1* (LASSO β = +0.263), sorafenib binding (−9.06 kcal/mol) may deplete thioredoxin-dependent antioxidant capacity upstream of *GPX4*, sensitizing high-risk tumors to ferroptotic stimuli. For *NQO1* and *NFE2L2*, no FDA-approved drug exceeded the −8.5 kcal/mol threshold, suggesting these targets may require purpose-designed small molecules.

Limitations include retrospective design, limited functional validation, BCR as surrogate endpoint, spatial resolution constraints (55 μm spots), potential batch effects, computational drug predictions requiring experimental validation, and lack of proteomic/metabolomic data. Treatment heterogeneity, including adjuvant radiation (8.7%) and systemic therapy received by a subset of patients, represents a potential confounding factor that could not be fully accounted for in this retrospective analysis. Additionally, the potential influence of patient age (range 41–78 years) on ferroptosis regulatory activity warrants investigation in future studies. Future directions include CRISPR screens, patient-derived organoids, xenograft models, clinical trials (sorafenib + abiraterone in mCRPC, immunotherapy + ferroptosis inducers in localized PRAD), mechanistic studies (AR–ferroptosis crosstalk, *NRF2* activation mechanisms), high-resolution spatial technologies (Visium HD, MERFISH), external validation in diverse populations, and therapeutic development of potent *GPX4* inhibitors.

## 4. Materials and Methods

### 4.1. Data Sources and Preprocessing

Bulk RNA-sequencing: RNA-seq data and clinical information for PRAD were obtained from TCGA (498 tumors, 52 normal). Raw counts were processed using DESeq2 v1.38.0 [44]. Gene identifiers were mapped from Ensembl IDs to HUGO symbols using org.Hs.eg.db v3.16.0 [45]. BCR was defined as PSA ≥ 0.2 ng/mL on two consecutive measurements [46]. Patient age at initial pathologic diagnosis ranged from 41 to 78 years (median 61 years, IQR 56–66 years, mean ± SD: 61.0 ± 6.8 years). All patients underwent radical prostatectomy as primary treatment. Neoadjuvant pharmaceutical therapy was administered in only 2 patients (0.4%). Adjuvant radiation therapy was received by 44 patients (8.7%); drug and radiation treatment records were available for 74 (14.7%) and 81 (16.1%) patients, respectively.

Single-cell RNA-sequencing: scRNA-seq data (68,322 cells, 24 donors) were obtained from GEO, profiled using 10× Genomics Chromium (10× Genomics, Pleasanton, CA, USA). Data were processed using Scanpy v1.9.1 [47].

Spatial transcriptomics: Spatial data (17 samples, 19,483 spots) were obtained from GEO using 10× Visium (10× Genomics, Pleasanton, CA, USA) (55 μm spot diameter). Data were processed using Scanpy and Squidpy v1.2.0 [48].

External validation cohorts: GSE116918 (*n* = 248, Affymetrix arrays; Thermo Fisher Scientific, Waltham, MA, USA) and GSE70769 (*n* = 94, Illumina BeadChip; Illumina, San Diego, CA, USA) were obtained from GEO with BCR outcomes. Expression data were normalized using RMA (Affymetrix) or quantile normalization (Illumina), and probe IDs were mapped to gene symbols using platform annotation files.

### 4.2. Ferroptosis Gene Set Curation

We compiled 80 ferroptosis genes from FerrDb v2 [49] (drivers, suppressors, markers) and literature curation [11,12,13,14,15,16,18,19,20] covering system Xc^−^-GSH-*GPX4* (glutathione peroxidase 4) axis, iron metabolism, lipid metabolism, and antioxidant defense. All gene sets were database-sourced with proper citations.

### 4.3. LASSO–Cox Prognostic Model

LASSO–Cox regression using glmnet v4.1–7 [50]. Risk score = Σ(βi × Expressioni). Performance: C-index, time-dependent ROC, calibration plots. External validation used identical risk score formula with available genes.

### 4.4. External Validation

Survival analysis: Kaplan–Meier analysis with log-rank test, univariate/multivariate Cox proportional hazards regression using survival v3.5–5 and survminer v0.4.9 [51,52].

### 4.5. Immune Landscape Analysis

Tumor microenvironment: ESTIMATE algorithm [53]. Immune deconvolution using 19 signatures from Charoentong et al. [54] with ssGSEA. Spearman correlation between ferroptosis genes and immune cell abundance.

### 4.6. Drug Sensitivity Analysis

Drug sensitivity: OncoPredict v0.2 [55] abiraterone, enzalutamide, RSL3, erastin, docetaxel, cabazitaxel, olaparib, anti-CTLA4, anti-PD1, anti-PDL1. Statistics: Wilcoxon test, Spearman correlation, Benjamini–Hochberg FDR.

### 4.7. Single-Cell RNA-Seq Analysis

Single-cell analysis: Ferroptosis scoring using scanpy.tl.score_genes() for five sub-pathways (ferroptosis_all, iron_metabolism, lipid_peroxidation, antioxidant_defense, GPX4_axis). Statistics: Mann–Whitney U test (cancer vs. benign), Kruskal–Wallis test (across cell types), Dunn’s test (post hoc), Benjamini–Hochberg FDR. Cell communication: 21 ligand–receptor pairs from CellChatDB [56]. TF activity: 10 TFs using MSigDB C3:TFT:GTRD target gene sets, AUCell algorithm, Spearman correlation with ferroptosis scores and ferroptosis resistance (inverse of ferroptosis score).

*NFE2L2* virtual KO simulation. In silico *NFE2L2* knockout was modeled by computing genome-wide Spearman correlations between *NFE2L2* expression and all other genes across all cells. Genes with |rho| > 0.1 and *p* < 0.05 were designated as regulatory targets. The KO perturbation delta for each cell was computed as the dot product of the cell’s gene expression vector with the *NFE2L2* correlation coefficients (masked to significant targets), representing predicted ferroptosis score change upon *NFE2L2* loss. Sub-pathway KO effects were computed separately for each ferroptosis axis. Cell-type aggregation used mean delta per major or minor cell type (minimum 5 cells). High- and low-responder cells were defined as the top and bottom quartile of KO delta scores, respectively, and differentially expressed markers were identified using the Mann–Whitney U approximation with Benjamini–Hochberg FDR correction.

Transcription factor activity: TF activity was estimated using single-sample Gene Set Enrichment Analysis (ssGSEA) with TF-target gene sets from the MSigDB C3:TFT:GTRD collection (736 TF regulons). We focused on 23 TFs with documented ferroptosis-regulatory roles, of which 10 had sufficient target gene overlap (≥5 genes detected in the scRNA-seq data) for reliable activity scoring: *NRF2* (*NFE2L2*), *AR*, *FOXA1*, *TP53*, *HIF1A*, *ATF4*, *BACH1*, *STAT3*, *MYC*, and *SP1*. Activity statistics (median, IQR) per cell type are provided in Supplementary Table S19.

### 4.8. In Situ Validation Using Spatial Transcriptomics

Spatial analysis: Per-spot ferroptosis scoring (same method as single-cell), spatial clustering (graph-based, k = 6 neighbors, Leiden algorithm), Kruskal–Wallis test across spatial clusters, visualization overlaid on H&E images.

### 4.9. Molecular Docking

Protein structures for *DHODH* (PDB: 1D3G), *NQO1* (PDB: 1D4A), *FSP1* (ferroptosis suppressor protein 1, encoded by *AIFM2*) (AlphaFold: Q9BRQ8), *TXNRD1*/*TrxR1* (AlphaFold: A0A182DWI3), *NOX4* (AlphaFold: Q9NPH5), *NFE2L2* (AlphaFold: Q16236), *GPX4* (PDB: 2OBI), and *xCT*/*SLC7A11* (AlphaFold: Q9UPY5) were prepared using AutoDockTools 1.5.7 (Center for Computational Structural Biology, Scripps Research, La Jolla, CA, USA): polar hydrogens added, Gasteiger charges assigned, non-polar hydrogens merged. FDA-approved drugs were obtained from DrugBank and prepared using OpenBabel 3.1.1 (open-source software, https://openbabel.org; 3D coordinates, protonation at pH 7.4). Docking was performed with AutoDock Vina 1.2.3 (Center for Computational Structural Biology, Scripps Research, La Jolla, CA, USA) using protein-specific search boxes (20–25 Å per side) centered on known or predicted binding pockets. Exhaustiveness was set to 8; top 9 poses were retained. Results were filtered for FDA-approved drugs and ranked by binding affinity (kcal/mol). Three-dimensional binding poses were visualized in PyMOL 2.5.

### 4.10. Statistical Analysis

Two significance criteria were applied: (1) statistical significance (*padj* < 0.05, Benjamini–Hochberg correction) identifies genes with reproducible expression differences, and (2) the additional fold-change filter (|log2FC| > 1) identifies genes with large effect sizes. In the main text, ‘differentially expressed’ refers to *padj* < 0.05 unless explicitly stated otherwise. The |log2FC| > 1 threshold was applied for volcano plot annotation and for the multi-modal integration criteria (Section 4.10).

Differential expression: DESeq2 v1.38.0 [44] with negative binomial GLM, median-of-ratios normalization, gene-wise dispersion estimation, Wald test, and Benjamini–Hochberg FDR correction. Significance: *padj* < 0.05, |log2FC| > 1.

Pathway analysis: GSVA v1.46.0 [57] with ssGSEA method on 234 pathways (50 Hallmark + 184 KEGG from MSigDB [58]). Differential activity: Wilcoxon test, Benjamini–Hochberg FDR, *padj* < 0.05.

Multi-modal integration: Cross-platform consensus gene identification. Criteria: bulk (*padj* < 0.05, |log2FC| > 1), scRNA (detected in >10% cells, significant cancer vs. benign), spatial (detected in >10% spots, spatial heterogeneity confirmed). Unified evidence table for 80 ferroptosis genes.

## 5. Conclusions

In summary, this comprehensive multi-omics study establishes ferroptosis dysregulation as a multi-scale phenomenon in PRAD. We identified a robust 16-gene prognostic signature and revealed that cancer cells evade ferroptosis through *NFE2L2*-associated transcriptional reprogramming and spatial compartmentalization. Our findings provide a computational rationale for combining ferroptosis inducers with androgen receptor inhibitors and immunotherapy, warranting further clinical translation.

Across the 80 ferroptosis-related genes analyzed, 71% (57/80) showed statistically significant expression changes in PRAD (*padj* < 0.05), although only 15% (12/80) reached the large effect size threshold of |log2FC| > 1, consistent with the relatively indolent transcriptional landscape of prostate adenocarcinoma.

## Data Availability

Publicly available datasets were analyzed in this study. These data can be found here: The Cancer Genome Atlas (TCGA-PRAD) at https://portal.gdc.cancer.gov (accessed on 9 May 2026) and the Gene Expression Omnibus (GEO) at https://www.ncbi.nlm.nih.gov/geo/ (accessed on 9 May 2026) (including accession numbers GSE116918 and GSE70769).

## Supplementary Materials

The following supporting information can be downloaded at: https://www.mdpi.com/article/10.3390/ijms27104448/s1.
