# Supplementary material for: NFE2L2-Associated Ferroptosis Resistance Reshapes the Tumor Immune Microenvironment and Guides Therapeutic Strategies in Prostate Cancer
Source: Int J Mol Sci. 2026 May 15;27(10):4448. doi: 10.3390/ijms27104448 (PMC13207672; doi:10.3390/ijms27104448)
Supplement: Supplementary file 1 [file ijms-27-04448-s001.zip › Supplemental Figures/S10_tf_violin_by_celltype/FigS10_tf_violin_by_celltype.pdf]

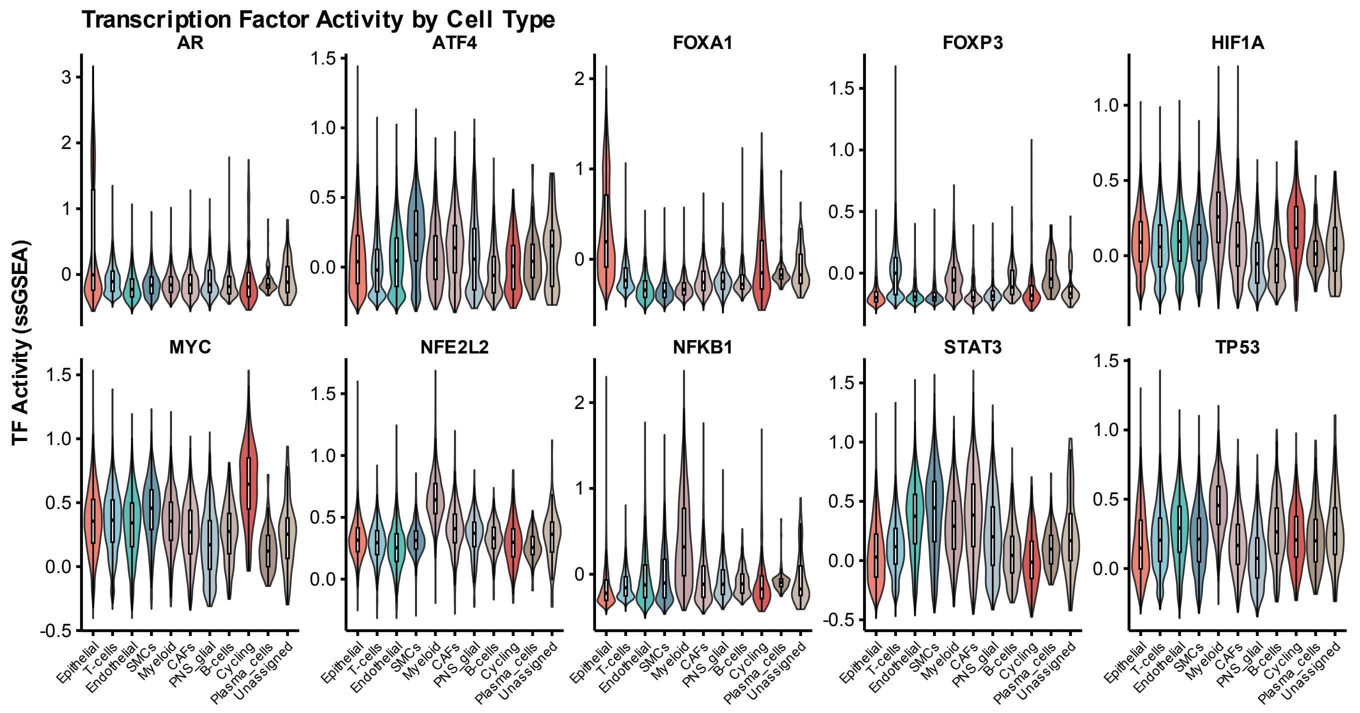
Transcription Factor Activity by Cell Type
TF Activity (ssGSEA)
AR
ATF4
FOXA1
FOXP3
HIF1A
MYC
NFE2L2
NFKB1
STAT3
TP53
Epithelial
T-cells
Endothelial
SMCs
Myeloid
CAFs
PNS_glial
B-cells
Cycling
Plasma_cells
Unassigned
