# Supplementary material for: NFE2L2-Associated Ferroptosis Resistance Reshapes the Tumor Immune Microenvironment and Guides Therapeutic Strategies in Prostate Cancer
Source: Int J Mol Sci. 2026 May 15;27(10):4448. doi: 10.3390/ijms27104448 (PMC13207672; doi:10.3390/ijms27104448)
Supplement: Supplementary file 1 [file ijms-27-04448-s001.zip › Supplemental Figures/S5_spatial_supplemental/S5B_multimodal_overview.pdf]

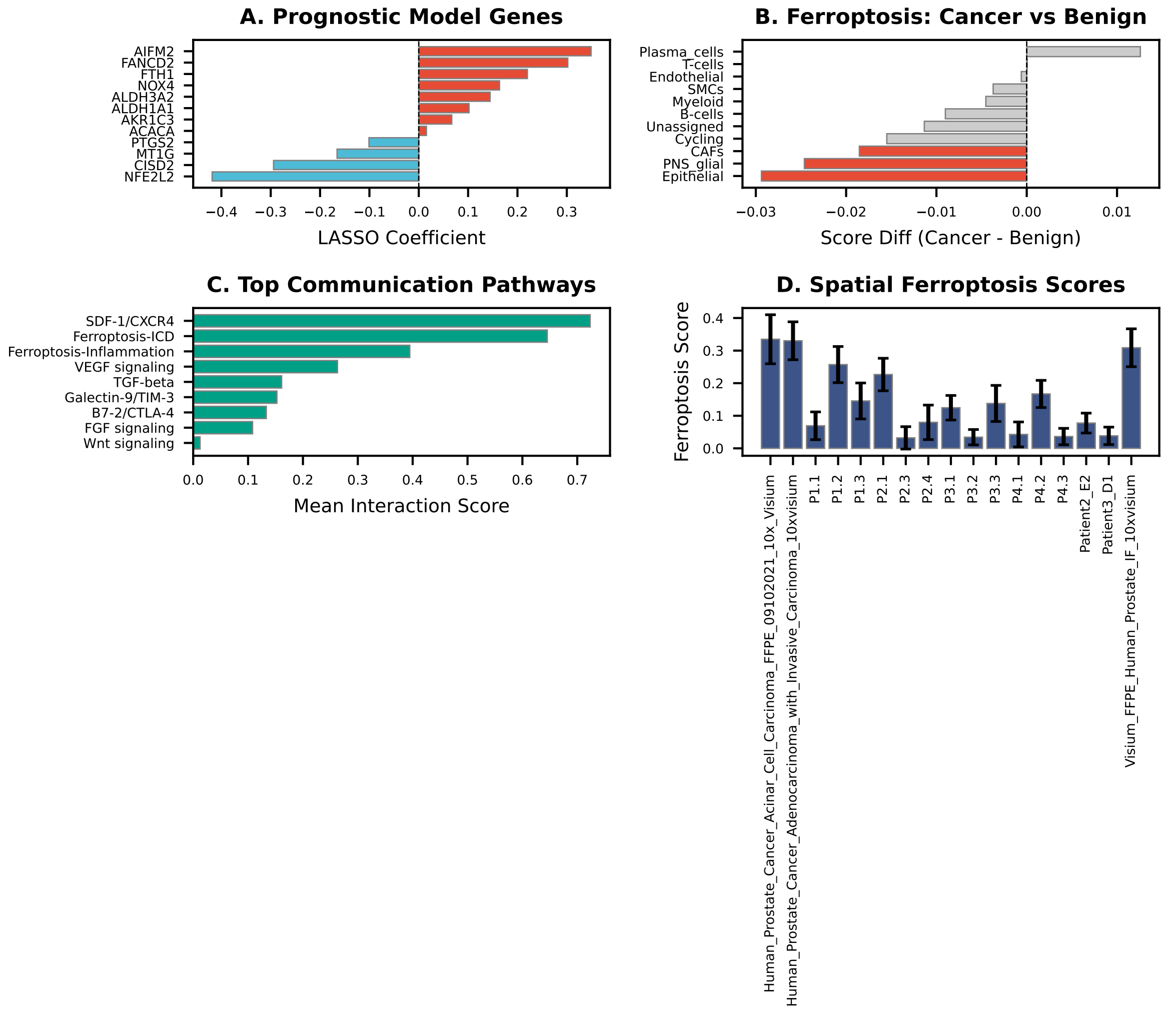
A. Prognostic Model Genes
AIFM2
FANCD2
FTH1
NOX4
ALDH3A2
ALDH1A1
AKR1C3
ACACA
PTGS2
MT1G
CISD2
NFE2L2
−0.4 −0.3 −0.2 −0.1 0.0 0.1 0.2 0.3
LASSO Coefficient
B. Ferroptosis: Cancer vs Benign
Plasma_cells
T-cells
Endothelial
SMCs
Myeloid
B-cells
Unassigned
Cycling
CAFs
PNS_glial
Epithelial
−0.03 −0.02 −0.01 0.00 0.01
Score Diff (Cancer - Benign)
C. Top Communication Pathways
SDF-1/CXCR4
Ferroptosis-ICD
Ferroptosis-Inflammation
VEGF signaling
TGF-beta
Galectin-9/TIM-3
B7-2/CTLA-4
FGF signaling
Wnt signaling
0.0 0.1 0.2 0.3 0.4 0.5 0.6 0.7
Mean Interaction Score
D. Spatial Ferroptosis Scores
Ferroptosis Score
0.0 0.1 0.2 0.3 0.4
Human_Prostate_Cancer_Acinar_Cell_Carcinoma_FFPE_09102021_10x_Visium
Human_Prostate_Cancer_Adenocarcinoma_with_Invasive_Carcinoma_10xvisium
P1.1
P1.2
P1.3
P2.1
P2.3
P2.4
P3.1
P3.2
P3.3
P4.1
P4.2
P4.3
Patient2_E2
Patient3_D1
Visium_FFPE_Human_Prostate_IF_10xvisium
