# Supplementary material for: NFE2L2-Associated Ferroptosis Resistance Reshapes the Tumor Immune Microenvironment and Guides Therapeutic Strategies in Prostate Cancer
Source: Int J Mol Sci. 2026 May 15;27(10):4448. doi: 10.3390/ijms27104448 (PMC13207672; doi:10.3390/ijms27104448)

Human_Prostate_Cancer_Acinar_Cell_Carcinoma_FFPE_09102021_10x_Visium: Ferroptosis Sub-pathways

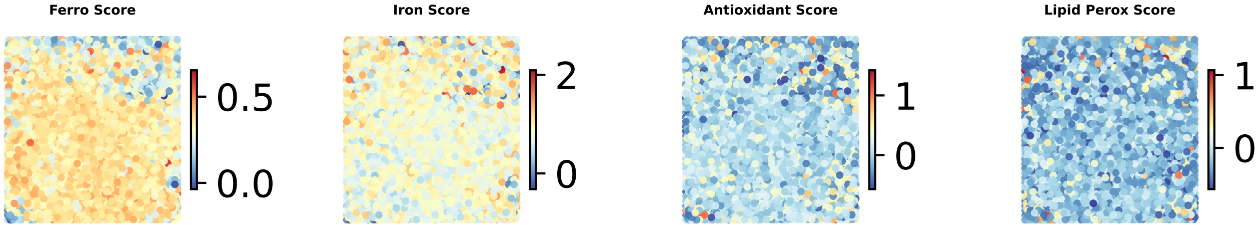

Supplement: Supplementary file 1 [file ijms-27-04448-s001.zip › Supplemental Figures/S5_spatial_supplemental/S5C_spatial_subpathways.pdf]
