# Supplementary figures and images for: NFE2L2-Associated Ferroptosis Resistance Reshapes the Tumor Immune Microenvironment and Guides Therapeutic Strategies in Prostate Cancer
Source: Int J Mol Sci. 2026 May 15;27(10):4448. doi: 10.3390/ijms27104448 (PMC13207672; doi:10.3390/ijms27104448)

## Consensus Clustering Metrics

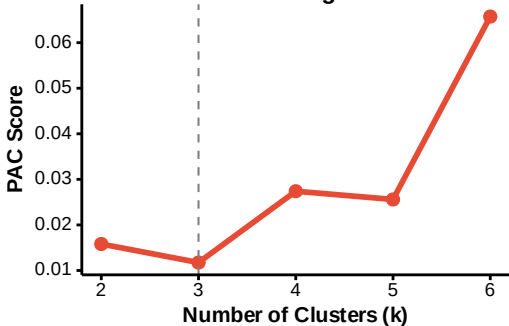

Supplement: Supplementary file 1 [file ijms-27-04448-s001.zip › Supplemental Figures/S1_consensus_clustering/S1A_cluster_metrics.pdf]

# Ferroptosis-Based Molecular Subtypes

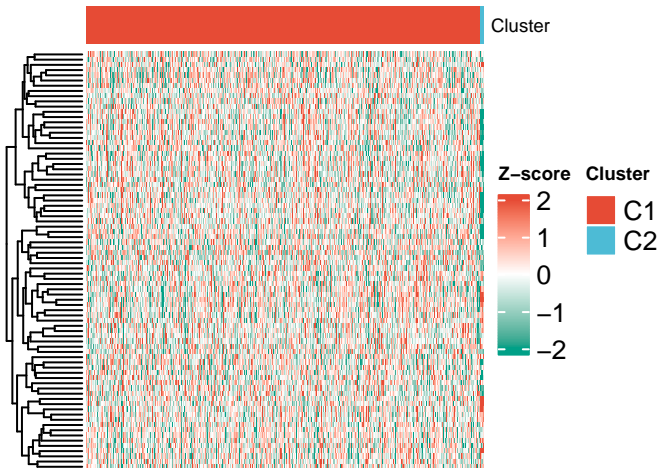

Supplement: Supplementary file 1 [file ijms-27-04448-s001.zip › Supplemental Figures/S1_consensus_clustering/S1B_cluster_heatmap.pdf]

# PCA of Ferroptosis Gene Expression

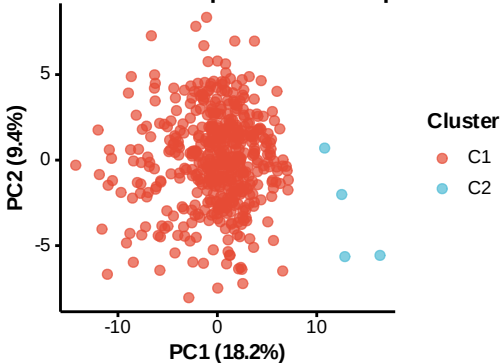

Supplement: Supplementary file 1 [file ijms-27-04448-s001.zip › Supplemental Figures/S1_consensus_clustering/S1C_cluster_PCA.pdf]

## Ferroptosis-Based Subtypes: BCR-free Survival

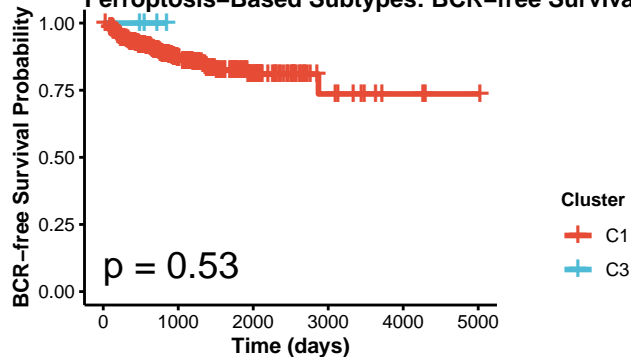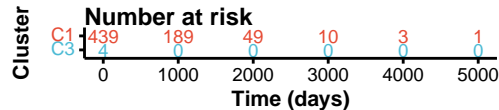

Supplement: Supplementary file 1 [file ijms-27-04448-s001.zip › Supplemental Figures/S2_survival_additional_genes/S2A_KM_clusters.pdf]

## ALDH3A2 : BCR-free Survival

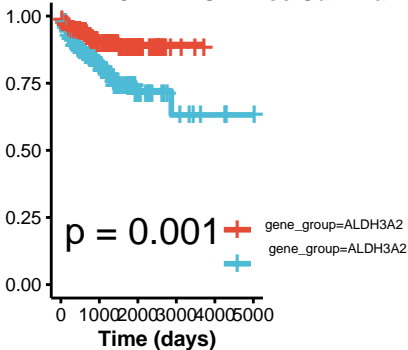

## Number at risk

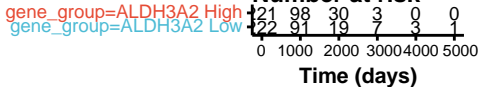

Supplement: Supplementary file 1 [file ijms-27-04448-s001.zip › Supplemental Figures/S2_survival_additional_genes/S2B_KM_ALDH3A2.pdf]

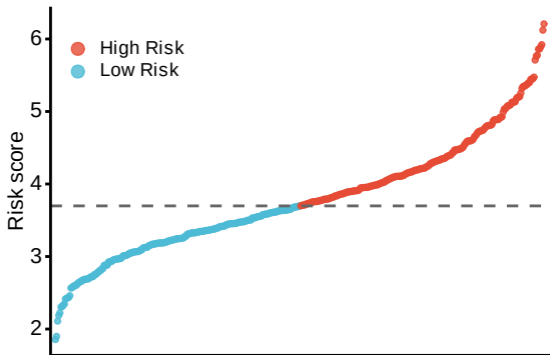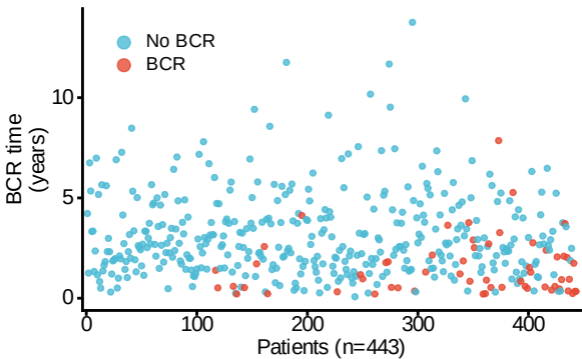

Supplement: Supplementary file 1 [file ijms-27-04448-s001.zip › Supplemental Figures/S2_survival_additional_genes/S2C risk_score_landscape.pdf]

# Multi-Omics Evidence for Ferroptosis Genes

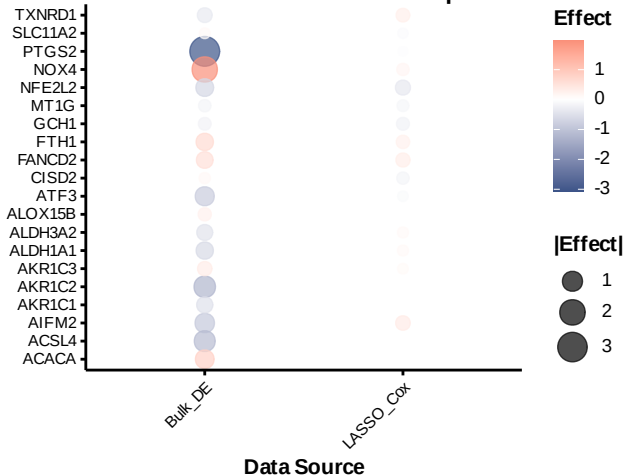

Supplement: Supplementary file 1 [file ijms-27-04448-s001.zip › Supplemental Figures/S3_multiomics_integration/S3A_multiomics_evidence_dotplot.pdf]

# Pathway Correlations with Ferroptosis

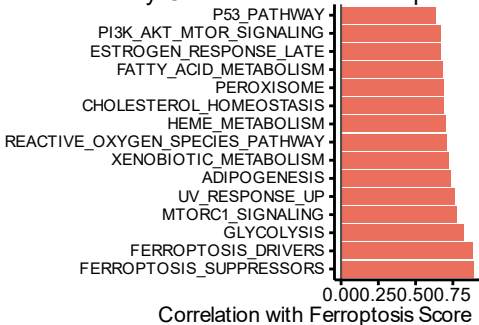

Positive

Supplement: Supplementary file 1 [file ijms-27-04448-s001.zip › Supplemental Figures/S3_multiomics_integration/S3B_ferroptosis_pathway_correlation.pdf]

# Malignancy Status

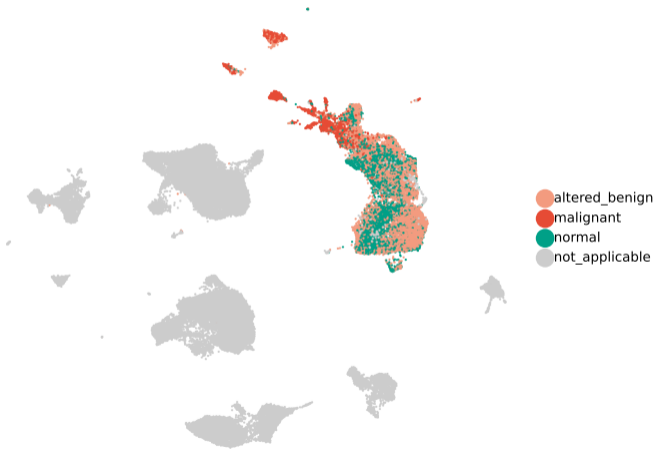

Supplement: Supplementary file 1 [file ijms-27-04448-s001.zip › Supplemental Figures/S4_scrna_supplemental/S4A_UMAP_malignancy.pdf]

**Ferroptosis Score: Cell Type x Tissue Type**

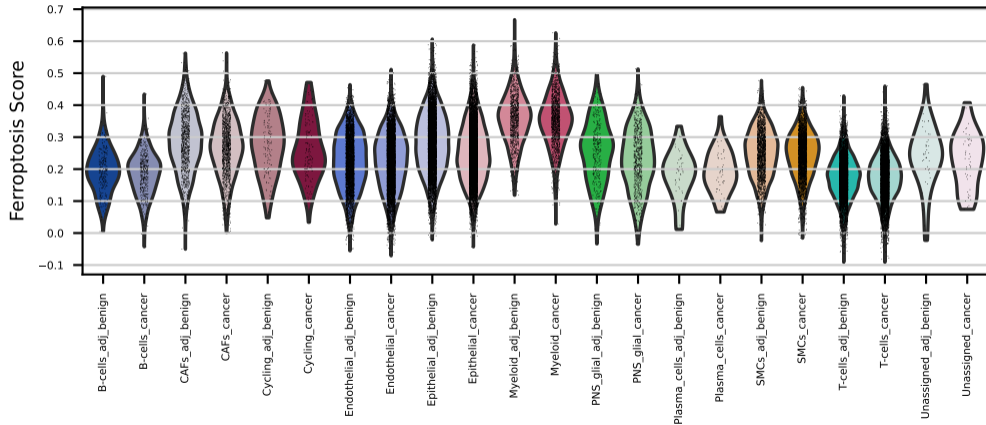

Supplement: Supplementary file 1 [file ijms-27-04448-s001.zip › Supplemental Figures/S4_scrna_supplemental/S4B_ferro_score_tissue.pdf]

# Ferroptosis Pathway Scores by Cell Type

celltype\_major\_v2

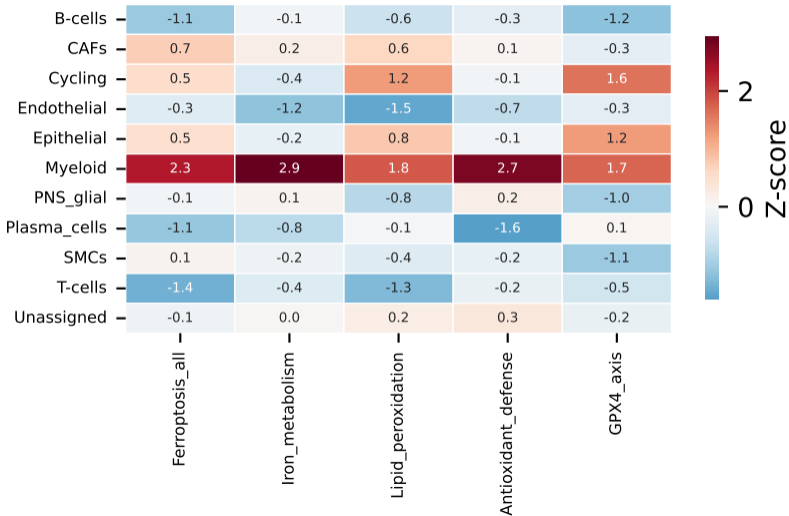

Supplement: Supplementary file 1 [file ijms-27-04448-s001.zip › Supplemental Figures/S4_scrna_supplemental/S4C_ferro_pathway_celltype.pdf]

# TF Activity by Cell Type (Z-scored)

celltype\_major\_v2

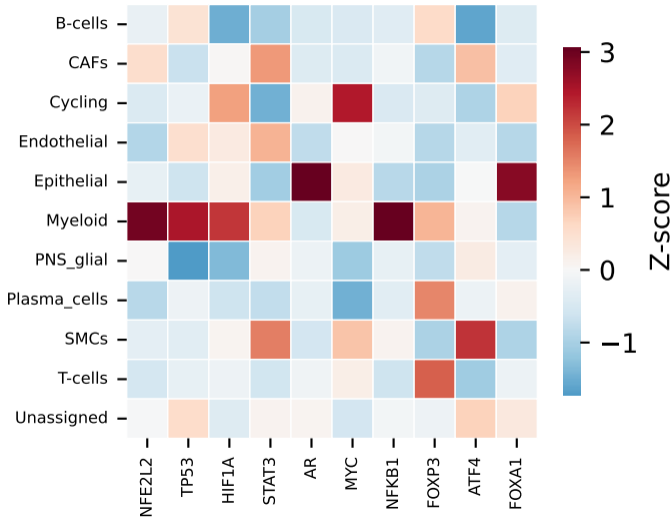

Supplement: Supplementary file 1 [file ijms-27-04448-s001.zip › Supplemental Figures/S4_scrna_supplemental/S4D_TF_celltype_heatmap.pdf]

# Ferroptosis Score by Spatial Cluster (All Samples)

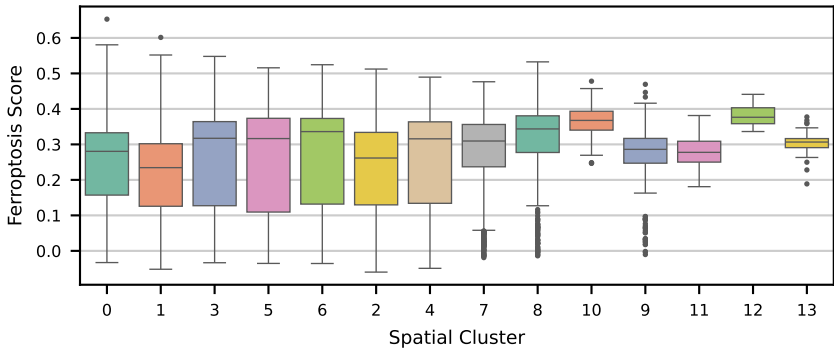

Supplement: Supplementary file 1 [file ijms-27-04448-s001.zip › Supplemental Figures/S5_spatial_supplemental/S5A_spatial_cluster_ferro.pdf]

# Top Ligand-Receptor Interactions

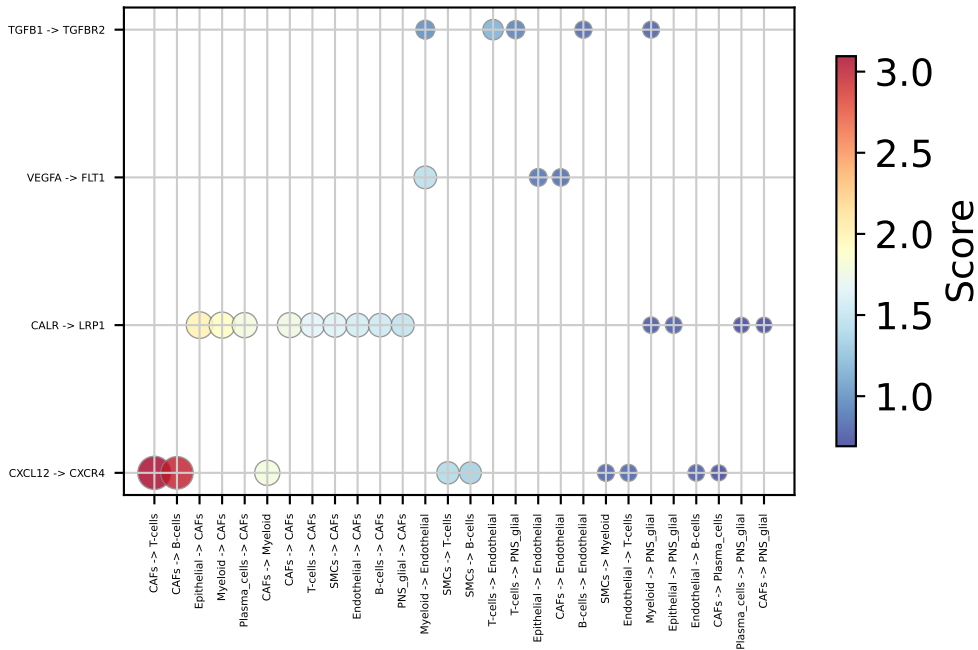

Supplement: Supplementary file 1 [file ijms-27-04448-s001.zip › Supplemental Figures/S6_cellcomm_pathways/S6A_top_LR_pairs.pdf]

# Integration: Ferroptosis Gene Evidence

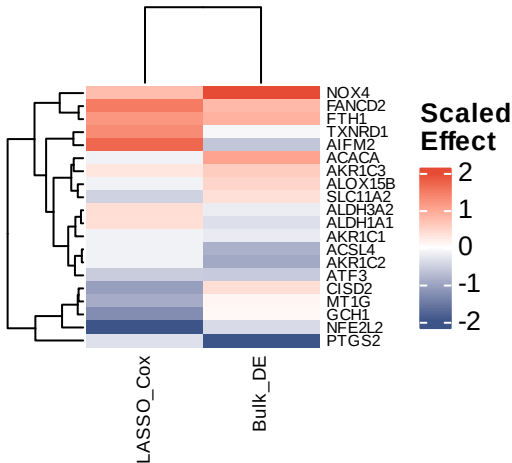

Supplement: Supplementary file 1 [file ijms-27-04448-s001.zip › Supplemental Figures/S6_cellcomm_pathways/S6B integration_evidence_heatmap.pdf]

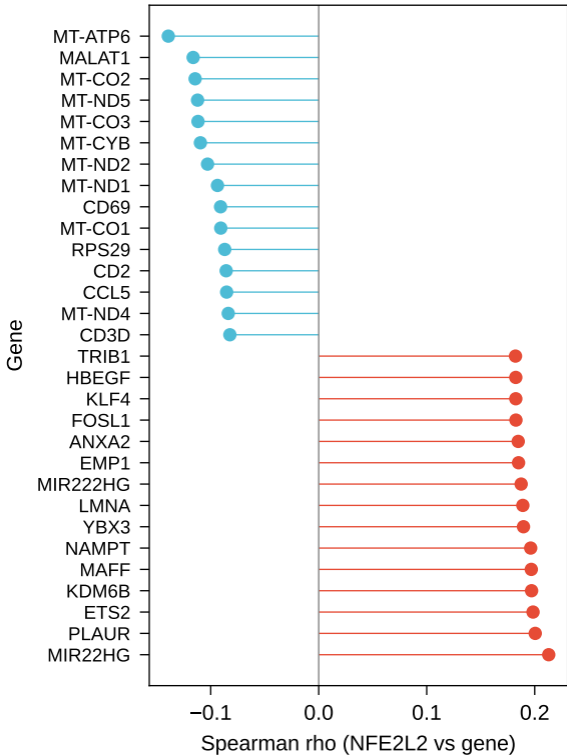

Supplement: Supplementary file 1 [file ijms-27-04448-s001.zip › Supplemental Figures/S7_nfe2l2_ko/S7A nfe2l2_grn_global_targets.pdf]

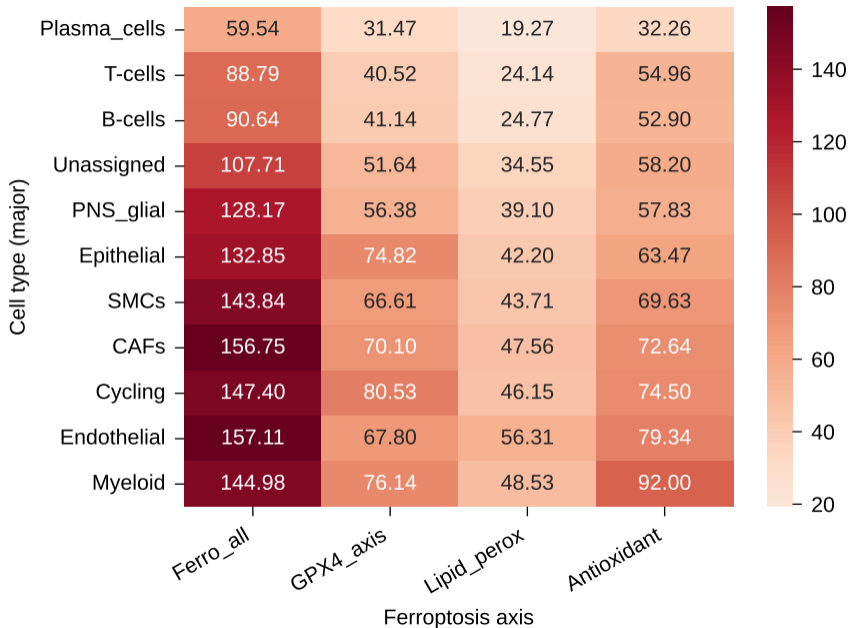

Supplement: Supplementary file 1 [file ijms-27-04448-s001.zip › Supplemental Figures/S7_nfe2l2_ko/S7B nfe2l2_ko_multiaxis_heatmap.pdf]

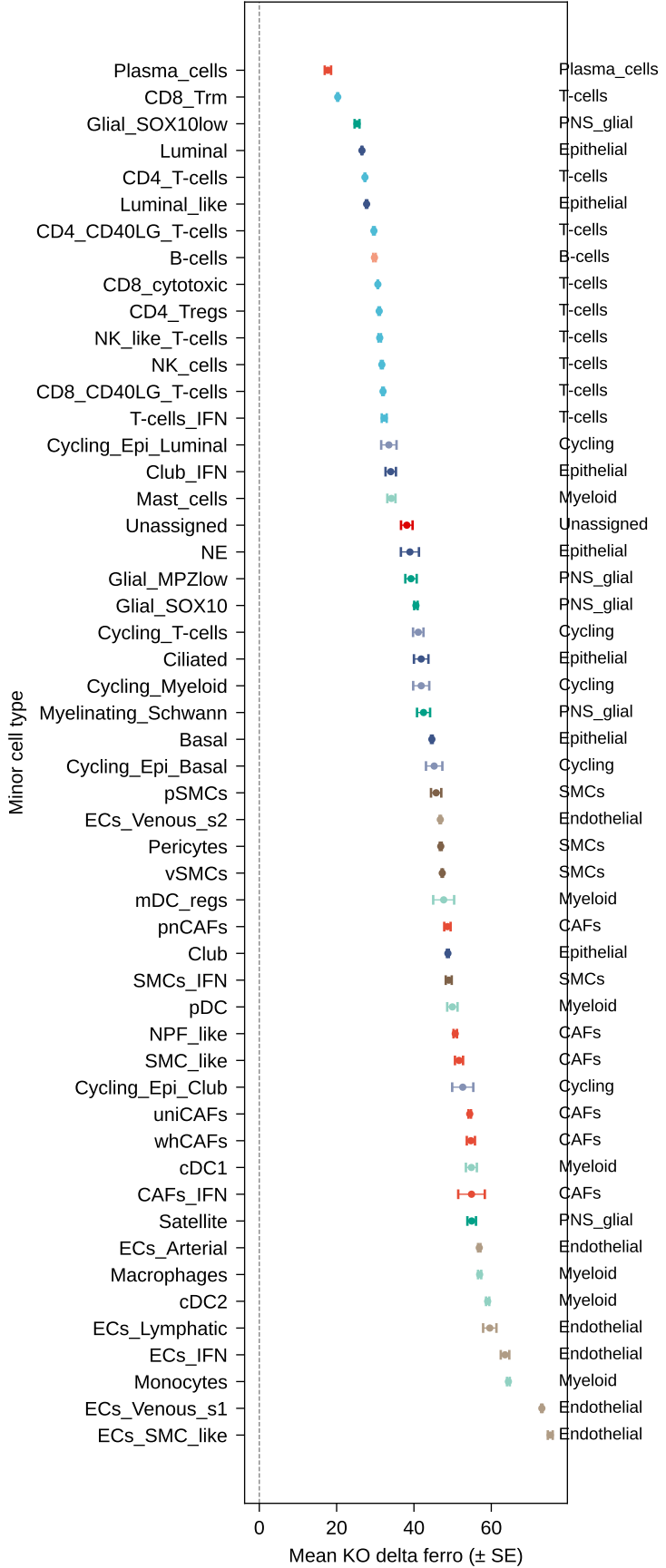

Supplement: Supplementary file 1 [file ijms-27-04448-s001.zip › Supplemental Figures/S7_nfe2l2_ko/S7C nfe2l2_ko_minor_celltype.pdf]

Gene

SH2D1A  
CD3G  
PYHIN1  
CD8B  
SCML4  
CD27  
GZMA  
CD3E  
GZMH  
ICOS  
THEMIS  
KLRC1  
CD8A  
CD2  
TRGC2  
CD247  
PRF1  
GPR174  
CD3D  
GPM6A

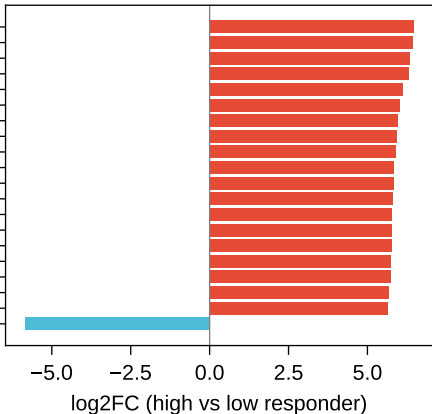

Supplement: Supplementary file 1 [file ijms-27-04448-s001.zip › Supplemental Figures/S7_nfe2l2_ko/S7D nfe2l2_ko_responder_markers.pdf]

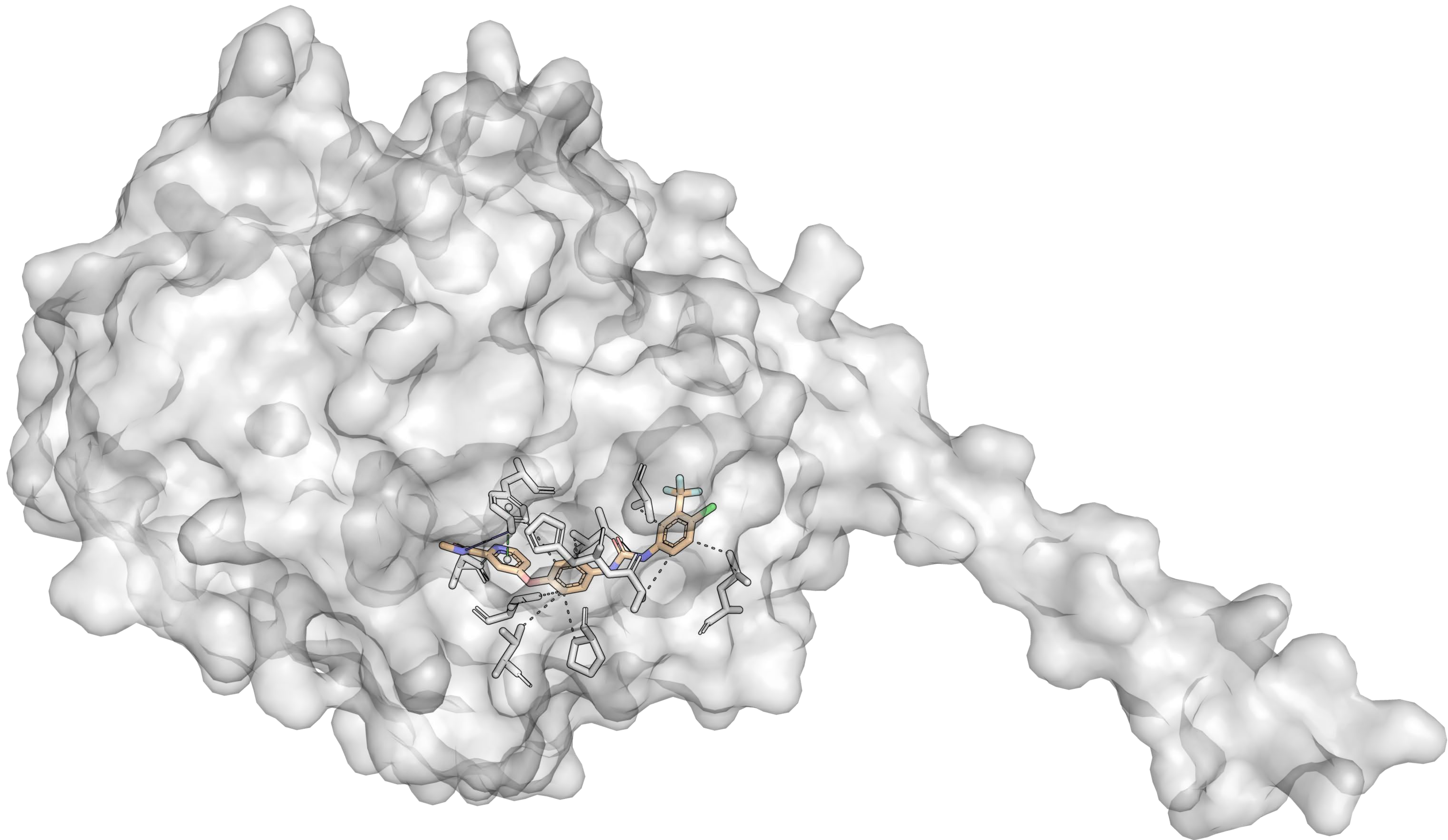

Supplement: Supplementary file 1 [file ijms-27-04448-s001.zip › Supplemental Figures/S8_sorafenib–DHODH/S8_sorafenib–DHODH.pdf]

# NFE2L2 correlation with classical NRF2 targets (68,322 cells)

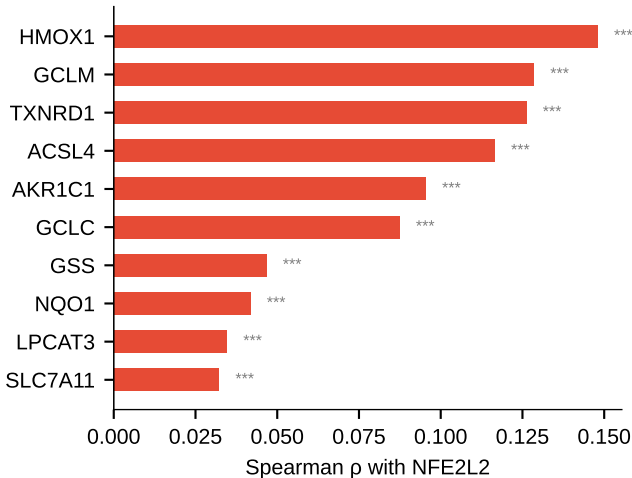

Supplement: Supplementary file 1 [file ijms-27-04448-s001.zip › Supplemental Figures/S9_NFE2L2_classical_targets_barplot/FigS9_NFE2L2_classical_targets_barplot.pdf]
